# Impact of energy dissipation on interface shapes and on rates for dewetting from liquid substrates

**DOI:** 10.1038/s41598-018-31418-1

**Published:** 2018-09-05

**Authors:** Dirk Peschka, Stefan Bommer, Sebastian Jachalski, Ralf Seemann, Barbara Wagner

**Affiliations:** 1 Weierstrass Institute for Applied Analysis and Stochastics, 10117 Berlin, Germany; 2 Experimental Physics, Saarland University, 66123 Saarbrücken, Germany

## Abstract

We revisit the fundamental problem of liquid-liquid dewetting and perform a detailed comparison of theoretical predictions based on thin-film models with experimental measurements obtained by atomic force microscopy. Specifically, we consider the dewetting of a liquid polystyrene layer from a liquid polymethyl methacrylate layer, where the thicknesses and the viscosities of both layers are similar. Using experimentally determined system parameters like viscosity and surface tension, an excellent agreement of experimentally and theoretically obtained rim profile shapes are obtained including the liquid-liquid interface and even dewetting rates. Our new energetic approach additionally allows to assess the physical importance of different contributions to the energy-dissipation mechanism, for which we analyze the local flow fields and the local dissipation rates. Using this approach, we explain why dewetting rates for liquid-liquid systems follow no universal power law, despite the fact that experimental velocities are almost constant. This is in contrast to dewetting scenarios on solid substrates and in contrast to previous results for liquid-liquid substrates using heuristic approaches.

## Introduction

**Figure 1:**
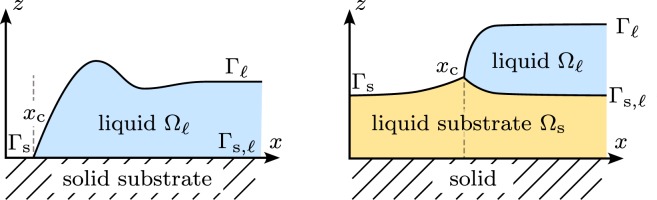
Fluid domain Ω(*t*), interfaces Γ_*i*_(*t*), and contact lines at *x*_c_(*t*) (left) for dewetting from a solid substrate where ${\rm{\Omega }}(t)={{\rm{\Omega }}}_{\ell }(t)$ and (right) for the considered situation of liquid dewetting from a liquid substrate, where ${\rm{\Omega }}(t)={{\rm{\Omega }}}_{\ell }(t)\cup {{\rm{\Omega }}}_{{\rm{s}}}(t)$.

The evolution of many physical systems is governed by thermodynamical or mechanical energetic principles^1–4^. Such principles are versatile instruments that allow the derivation of underlying physical equations^5^. For flows of incompressible liquids, energy-dissipation principles are known for a long time^6–8^. In particular for thin-film flows, the great success in the quantitative understanding of viscous flows with contact-line motion has supplied a universal tool that enables the nano- and microstructuring and functionalization of surfaces, but moreover allows to relate flow patterns with liquid properties and substrate chemistry^9^. Typical phenomena governed by such principles are the dewetting of liquids from solid substrates and from liquid substrates, or general wetting and spreading phenomena^10–14^, where the balance of the decline of energy and the dissipation $\dot{ {\mathcal E} }=-\,{\mathscr{D}}\le 0$ can be used to derive power-law rates for the velocity of moving contact lines, see Fig. 1.

When such a power-law rate *x*_c_ ~ *t*^*β*^ exists, its exponent *β* reveals the dominant physical effect, *e*.*g*., gravity, surface tension, viscous dissipation in bulk and on interfaces, and the geometry of the problem^15^. One basic assumption behind such rate estimates is that there exists a simple relationship between the rate of change of the energy and the shape of the time-dependent domain ${\rm{\Omega }}(t)\subset {{\mathbb{R}}}^{3}$ occupied by the liquid layer, often also including assertions about the self-similarity of the evolution. For instance, for a large class of free boundary problems where a liquid $(\ell )$ dewetts from a substrate (s) with a straight contact line, the change of surface energy can be approximated by

1 $$\dot{ {\mathcal E} }=\tfrac{{\rm{d}}}{{\rm{d}}t}({\gamma }_{{\rm{s}}}|{{\rm{\Gamma }}}_{{\rm{s}}}|+{\gamma }_{s,\ell }|{{\rm{\Gamma }}}_{s,\ell }|+{\gamma }_{\ell }|{{\rm{\Gamma }}}_{\ell }|)\approx S\times {\dot{x}}_{{\rm{c}}},$$

where $S={\gamma }_{{\rm{s}}}-({\gamma }_{{\rm{s}},\ell }+{\gamma }_{\ell }) < 0$ is the spreading coefficient of the system constructed from the corresponding surface- and interface-tensions *γ*_*α*_ of interfaces Γ_*α*_ with surface area |Γ_*α*_| and ${\dot{x}}_{{\rm{c}}}$ is the contact line velocity. However, it is challenging or even impossible to find a general and similarly simple closed-form approximation for the energy-dissipation rate

2 $${\mathscr{D}}({\bf{u}})={\int }_{{\rm{\Omega }}}\,{\boldsymbol{\tau }}({\bf{u}}):\nabla {\bf{u}}\,{\rm{d}}x\,{\rm{d}}y\,{\rm{d}}z,$$

since the flow field ${\bf{u}}:{\rm{\Omega }}\to {{\mathbb{R}}}^{3}$ and the corresponding shear stress ***τ***(**u**) have a complicated local structure that depends on fine details of the shape of Ω. Therefore, one requires a deeper understanding of the specific dissipation mechanisms and of the domain evolution in order to understand the dynamics of the corresponding processes.

In the pioneering works for liquid-liquid dewetting by Joanny^13^ and Brochard-Wyart *et al*.^14^ dewetting rates for small equilibrium contact angles and for limiting regimes of the liquid-liquid viscosity ratios were predicted. While both works combine valid hydrodynamical and dissipation arguments to derive expressions for contact line velocities, the impact of non-trivial interface shapes on the flow and dissipation remained unclear. Since then, many theoretical studies are concerned with the derivation of appropriate thin-film models to study the long-time morphological evolution of the liquid layers. Apart from investigations into stationary states and how they are approached^16,17^, a number of studies focussed on modes of instability in liquid-liquid dewetting using stability analysis and numerical simulations of the thin-film models^18–22^ even with additional surfactants^23^.

On the experimental side dewetting rates and morphologies for liquid-liquid model systems such as polystyrene on polymethyl methacrylate have been investigated systematically by Krausch *et al*.^24,25^ by varying the heights and viscosities of the liquid layers. Similar experimental studies were performed by Pan *et al*.^26^ for further layer viscosities and heights. However, the shapes observed by Krausch *et al*.^25^ differ considerably from the empirical predictions used to derive dewetting rates^13,14^, which were found to be constant.

To the best of our knowledge, fundamental dynamic properties like dewetting rates have not been settled up to now. The main reason for this is certainly the absence of theoretical confirmations for the observed shapes of dewetting rims, which then might help to understand the mechanisms behind certain dissipation balances and dewetting rates. Additionally, a quantitative study also requires the key parameters of the experimental system, *i*.*e*., surface tensions, viscosities, and layer thicknesses, to be determined sufficiently precise. The focus of this study is thus to supply a quantitative understanding of the dewetting mechanics by detailed comparisons of experimentally obtained rim shapes, their evolution, and their dewetting dynamics with those computed from thin-film equations. Additionally, we examine the underlying mechanisms by discussing flow fields and energy dissipation mechanisms.

## Experimental and Theoretical Methods

**Figure 2:**
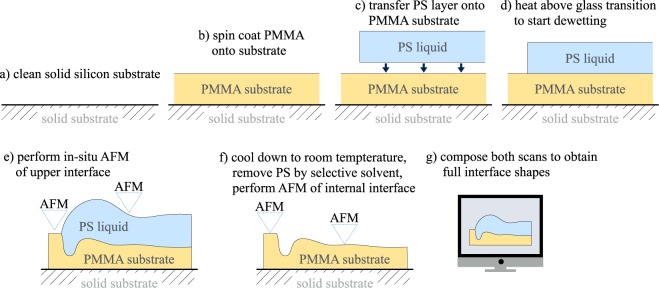
(**a**–**d**) Experimental preparation of thin-film bilayer system and (**e**,**f**) AFM measurement and (**g**) postprocessing.

As a model system we consider a layer of viscous liquid polystyrene (PS) ${{\rm{\Omega }}}_{\ell }$ above a viscous liquid substrate consisting of polymethyl methacrylate (PMMA) Ω_s_. Both liquids are immiscible and the total liquid domain is ${\rm{\Omega }}={{\rm{\Omega }}}_{\ell }\cup {{\rm{\Omega }}}_{{\rm{s}}}$ and depends on time. Using the functions ${h}_{\ell }$, *h*_s_ to represent the thickness of fluid and substrate layer, one can parametrize the domains at time *t*

3 $$\begin{array}{rcl}{{\rm{\Omega }}}_{{\rm{s}}}(t) & = & \{(x,y,z)\in {{\mathbb{R}}}^{3}:\,0 < z < {h}_{{\rm{s}}}(t,x,y)\},\\ {{\rm{\Omega }}}_{\ell }(t) & = & \{(x,y,z)\in {{\mathbb{R}}}^{3}:{h}_{{\rm{s}}}(t,x,y) < z < {h}_{{\rm{s}}}(t,x,y)+{h}_{\ell }(t,x,y)\},\end{array}$$

and the corresponding free surfaces and internal interfaces are

4 $$\begin{array}{rcl}{{\rm{\Gamma }}}_{{\rm{s}}}(t) & = & \{(x,y,z)\in {{\mathbb{R}}}^{3}:(z={h}_{{\rm{s}}}(t,x,y))\cap ({h}_{\ell }(t,x,y)=\mathrm{0)\},}\\ {{\rm{\Gamma }}}_{\ell }(t) & = & \{(x,y,z)\in {{\mathbb{R}}}^{3}:(z={h}_{{\rm{s}}}(t,x,y)+{h}_{\ell }(t,x,y))\cap ({h}_{\ell }(t,x,y) > \mathrm{0)\},}\\ {{\rm{\Gamma }}}_{{\rm{s}},\ell }(t) & = & \{(x,y,z)\in {{\mathbb{R}}}^{3}:(z={h}_{{\rm{s}}}(t,x,y))\cap ({h}_{\ell }(t,x,y) > \mathrm{0)\},}\end{array}$$

as indicated in Fig. 1. Initially, at room temperature both polymer layers are in a glassy state. The substrate layer (PMMA) has a constant thickness ${h}_{{\rm{s}}}(t=0,x,y)={\bar{h}}_{{\rm{s}}}$ and is supported by a solid silicon wafer at *z* = 0. The contour of the upper liquid layer (PS) is piecewise constant with an almost rectangular edge ${h}_{\ell }(t=0,x,y)={\bar{h}}_{\ell }$ for *x* > *x*_c_(*t* = 0) and ${h}_{\ell }(t=0,x,y)=0$ for *x* ≤ *x*_c_(*t* = 0), which is generated by the preparation process. During the evolution the domain shape Ω will remain approximately translational invariant in direction parallel to the straight three phase contact line, so that it is sufficient to consider cross sections of the domain and of the velocity fields in the *x*-*z*-plane. The layered polymer system was prepared using standard thin film preparation techniques^17^ as shown in Fig. 2. In a first step, the underlying liquid PMMA substrate is directly spun from a toluene solution onto a silicon substrate that was previously cleaned with Piranha etch. Simultaneously, the upper PS layer is spun from a toluene solution onto freshly cleaved muscovite mica. In a second step, the PS layer is transferred onto the surface of ultra clean water and picked up from there with the PMMA coated silicon substrate. During the transfer of the thin PS layer onto the water, the PS layer ruptures into smaller pieces, which are subsequently transferred onto the spin coated PMMA layer. Straight boundaries of these patches that are sufficiently remote not to be disturbed from neighboring patches are selected to observe the dewetting process. The cross section of these patches are almost ideal rectangular steps and thereby correspond to the start configurations at *t* = 0 introduced above. The typical film-thicknesses ${\bar{h}}_{\ell },{\bar{h}}_{{\rm{s}}}$ used in our dewetting experiments range from 45 nm to 250 nm and we performed experiments for various ratios ${\bar{h}}_{\ell }:{\bar{h}}_{{\rm{s}}}$.

**Figure 3:**
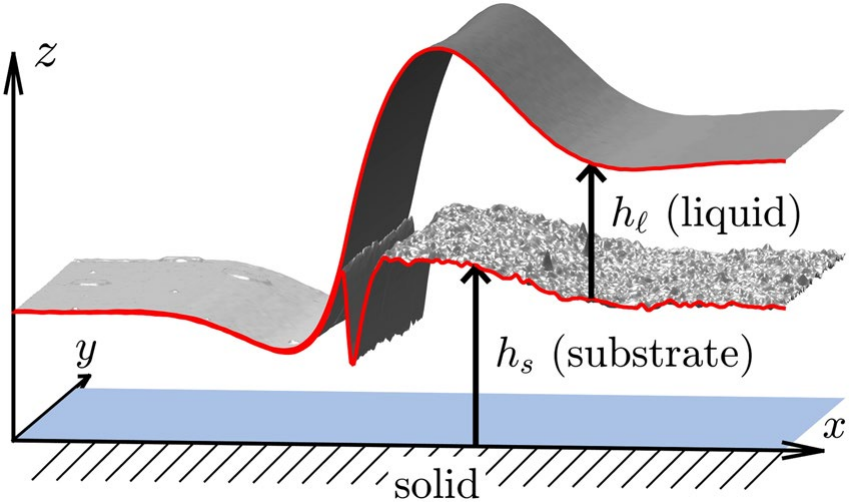
Measured 3-*D* shape of a dewetting rim composed of AFM scans of PMMA-air, PS-air, and PS-PMMA interface with liquid PS layer ${\bar{h}}_{\ell }=\mathrm{(125}\pm \mathrm{5)}\,{\rm{nm}}$ and liquid PMMA substrate ${\bar{h}}_{{\rm{s}}}=\mathrm{(125}\pm \mathrm{5)}\,{\rm{nm}}$ after dewetting for 24 h at *T* = 140 °C.

To remove potential nanoscopic air bubbles that might have been been trapped between the PMMA and the PS layer during the transfer process, the samples were allowed to set after preparation for at least 24 h. The dewetting process is then started by heating the materials above the glass transition temperature and monitored by *in situ* atomic force microscopy (AFM). The dewetting experiments were conducted at a temperature of *T* = 140 °C. The shape of the PS-air and PMMA-air interface can be determined *in situ* using AFM in soft tapping mode. Quenching the sample to room temperature the shape of the buried PS-PMMA interface can be additionally determined by AFM in tapping mode after stripping the upper PS layer with a selective solvent (cyclohexane, Sigma Aldrich)^17^. The full shape of all polymer interfaces is obtained by composing PS-air, PMMA-air and PS-PMMA surfaces, a procedure which generates shapes as shown in Fig. 3. This composition of the 3-*D* image requires rotation, shift and tilt of upper and lower AFM scan as postprocessing for a perfect match. The contact line is also aligned parallel to the *y*-axis, so that cross sections can be averaged over a few scan lines in the *y*-direction. Both polymers were purchased from Polymer Standard Service Mainz (PSS-Mainz, Germany) with polydispersity of *M*_*w*_/*M*_*n*_ = 1.05 and molecular weights of *M*_*w*_ = 64 kg/mol and *M*_*w*_ = 9.9 kg/mol for PS and PMMA, respectively. The glass transition temperatures are *T*_g_ = 100 ± 5 °C for PS(64 k) and *T*_g_ = 115 ± 5 °C for PMMA(9.9 k). The viscosities of these polymers at *T* = 140 °C were determined for PS as ${\mu }_{\ell }\approx 700\,{\rm{kPa}}\,{\rm{s}}$ and for PMMA as *μ*_s_ ≈ 700 kPas applying the method of self-similarity profiles of stepped polymer films^27,28^. Using stationary droplet profiles^17^ we experimentally determined the involved surface tensions to ${\gamma }_{\ell }=31.5\pm 0.2\,{\rm{mN}}/{\rm{m}}$, *γ*_s_ = 32 ± 0.2 mN/m and ${\gamma }_{{\rm{s}},\ell }=1.2\pm 0.2\,{\rm{mN}}/{\rm{m}}$, compatible with values reported in literature^29^. Using the Neumann triangle construction

$$\begin{array}{rcl}{\gamma }_{\ell }\,\cos \,{\vartheta }_{1}+{\gamma }_{{\rm{s}},\ell }\,\cos \,{\vartheta }_{2} & = & {\gamma }_{{\rm{s}}},\\ {\gamma }_{\ell }\,\sin \,{\vartheta }_{1}-{\gamma }_{{\rm{s}},\ell }\,\sin \,{\vartheta }_{2} & = & \mathrm{0,}\end{array}$$

these values correspond to contact angles of the PS-air interface with respect to the undisturbed PMMA-air interface *ϑ*_1_ = 2° and of the PS-PMMA interface with respect to the undisturbed PMMA-air interface of *ϑ*_2_ = 64° and imply a spreading coefficient of *S* = (−0.7 ± 0.6)mN/m. However, a quantitative agreement of experimental measurement and numerical simulations concerning the temporal evolution and concerning the observed interface shapes requires setting the viscosities to ${\mu }_{\ell }={\mu }_{{\rm{s}}}=1100\,{\rm{kPa}}\,{\rm{s}}$ and the spreading coefficient to *S* = −1.5 mN/m. These determined parameters are in the range of values reported in literature and compatible with the experimental values. We use these parameters consistently for all film thicknesses. The parameters ${\mu }_{\ell }:{\mu }_{{\rm{s}}}\sim 1$ and ${\bar{h}}_{\ell }:{\bar{h}}_{{\rm{s}}}\sim 1$ suggest, that initially dissipation in the substrate and in the liquid are of the same order.

The fluid flow at time *t* is described by the continuous velocity field ${\bf{u}}:{\rm{\Omega }}\to {{\mathbb{R}}}^{3}$ and satisfies a no-slip condition at the solid substrate, *i*.*e*., **u**(*x*, *y*, *z* = 0) = **0**. In particular, this flow is incompressible $\nabla \cdot {\bf{u}}=0$ and according to (2) it dissipates the energy

5 $${\mathscr{D}}({\bf{u}})={\int }_{{\rm{\Omega }}}\,\tau ({\bf{u}}):\nabla {\bf{u}}\,{\rm{d}}x\,{\rm{d}}y\,{\rm{d}}z={\int }_{{{\rm{\Omega }}}_{\ell }}\,{{\boldsymbol{\tau }}}_{\ell }({{\bf{u}}}_{\ell }):\nabla {{\bf{u}}}_{\ell }\,{\rm{d}}x\,{\rm{d}}y\,{\rm{d}}z+{\int }_{{{\rm{\Omega }}}_{{\rm{s}}}}\,{{\boldsymbol{\tau }}}_{{\rm{s}}}({{\bf{u}}}_{{\rm{s}}}):\nabla {{\bf{u}}}_{{\rm{s}}}\,{\rm{d}}x\,{\rm{d}}y\,{\rm{d}}z,$$

where we introduced the shear stress for a two-phase system consisting of Newtonian liquids as

6 $${\boldsymbol{\tau }}({\bf{u}})=\mu (\nabla {\bf{u}}+\nabla {{\bf{u}}}^{{\rm{{\rm T}}}}),\,{\rm{where}}\,\mu (x,y,z)=(\begin{array}{cc}{\mu }_{\ell } & (x,y,z)\in {{\rm{\Omega }}}_{\ell }\\ {\mu }_{{\rm{s}}} & (x,y,z)\in {{\rm{\Omega }}}_{{\rm{s}}}\end{array},$$

and denote with **u**_*i*_ and ***τ***_*i*_ the restriction of **u** and ***τ*** to Ω_*i*_ for $i\in \{{\rm{s}},\ell \}$ representing the substrate or the liquid. By construction, we have ${{\bf{u}}}_{{\rm{s}}}={{\bf{u}}}_{\ell }$ on ${{\rm{\Gamma }}}_{{\rm{s}},\ell }$ and **u**_s_ = 0 at *z* = 0.

From the experimental dewetting rates discussed later one can anticipate shear rates in the PS film of $\dot{\gamma }\approx 3\cdot {10}^{-4}$ s^−1^. Using the fitted polymer viscosities and the shear modulus of *G*_PS_ = 0.2 MPa for PS^30^ and *G*_PMMA_ = 3 MPa for PMMA^31^, the relaxation time of the polymers *τ*_PS_ = *μ*_PS_/*G*_PS_ ≈ 4 s and *τ*_PMMA_ ≈ 0.2 s can be calculated. The resultant Weissenberg numbers ${\rm{Wi}}=\tau \,\dot{\gamma }$ are ${{\rm{Wi}}}_{{\rm{PMMA}}}\approx {10}^{-4}\ll 1$ and ${{\rm{Wi}}}_{{\rm{PS}}}\approx {10}^{-3}\ll 1$, so that viscoelastic effects can be safely neglected^32^ and the treatment of the PS-PMMA system as Newtonian liquids is justified.

The energy from (1) is defined ${\mathcal E} ={\gamma }_{{\rm{s}}}|{{\rm{\Gamma }}}_{{\rm{s}}}|+{\gamma }_{s,\ell }|{{\rm{\Gamma }}}_{s,\ell }|+{\gamma }_{\ell }|{{\rm{\Gamma }}}_{\ell }|$, where we can use the representation via ${h}_{\ell }$, *h*_s_ to write

7 $$\begin{array}{rcl}|{{\rm{\Gamma }}}_{{\rm{s}},\ell }| & = & {\int }_{\omega }\,\sqrt{1+|\nabla {h}_{{\rm{s}}}{|}^{2}}\,{\rm{d}}x\,{\rm{d}}y,\\ |{{\rm{\Gamma }}}_{\ell }| & = & {\int }_{\omega }\,\sqrt{1+|\nabla ({h}_{{\rm{s}}}+{h}_{\ell }{)|}^{2}}\,{\rm{d}}x\,{\rm{d}}y,\\ |{{\rm{\Gamma }}}_{{\rm{s}}}| & = & {\int }_{{{\mathbb{R}}}^{2}\backslash \omega }\,\sqrt{1+|\nabla {h}_{{\rm{s}}}{|}^{2}}\,{\rm{d}}x\,{\rm{d}}y,\end{array}$$

where we introduced the wetted area as the time-dependent support set of ${h}_{\ell }$ using

8 $$\omega (t)=\{(x,y)\in {{\mathbb{R}}}^{2}:{h}_{\ell }(t,x,y) > \mathrm{0\}}\subset {{\mathbb{R}}}^{2}\mathrm{.}$$

The apparent problem of an infinite energy ${\mathcal E}$ in (7) for unbounded *ω* in (8) can be circumvented by formally restricting the whole domain to a sufficiently big finite box. For given dissipation ${\mathscr{D}}$ and energy ${\mathcal E}$ it is basically known since the works of Helmholtz^6^ and Rayleigh^7^, that the evolution of the system is given by a minimal dissipation principle

9 $${\bf{u}}=\mathop{{\rm{argmin}}}\limits_{{\bf{v}}}\,(\frac{1}{2}{\mathscr{D}}({\bf{v}})+\langle {\rm{D}} {\mathcal E} ,{\bf{v}}\rangle ),$$

or equivalently upon differentiation by the weak formulation *a*(**u**, **v**) = *f*(**v**) for all divergence-free **v**, where using (5) we have the bilinear form $a({\bf{u}},{\bf{v}})={\int }_{{\rm{\Omega }}}\,{\boldsymbol{\tau }}({\bf{u}}):\nabla {\bf{v}}\,{\rm{d}}x\,{\rm{d}}y\,{\rm{d}}z$ and the linear functional $f({\bf{v}})=-\,\langle {\rm{D}} {\mathcal E} ,{\bf{v}}\rangle$. The computation of *f* requires the formal variation of surface measures |Γ_*i*_| with respect to perturbations of the surface by a flow **v**. This variation involves the Laplace-Beltrami operator^33^ and for our energy ${\mathcal E}$ can be expressed as

10 $$f({\bf{v}})=-\,\sum _{i\in \{\ell ,{\rm{s}},s\ell \}}\,{\gamma }_{i}\,{\int }_{{{\rm{\Gamma }}}_{i}}\,\bar{\nabla }{\rm{id}}:\bar{\nabla }{\bf{v}}\,{\rm{d}}a=\sum _{i}\,2{\gamma }_{i}\,{\int }_{{{\rm{\Gamma }}}_{i}}\,\kappa {\boldsymbol{\nu }}\cdot {\bf{v}}\,{\rm{d}}a-{\int }_{\partial {\rm{\Gamma }}}\,{{\bf{f}}}_{{\rm{cl}}}\cdot {\bf{v}}\,{\rm{d}}s,$$

where *κ****ν*** is the mean curvature vector on ${{\rm{\Gamma }}}_{\ell }$ and $\bar{\nabla }$ the tangential gradient. The Young force ${{\bf{f}}}_{{\rm{cl}}}={\sum }_{i}\,{\gamma }_{i}{{\boldsymbol{\nu }}}_{i}$ appears at the contact line ∂Γ and is generated by performing integration-by-parts using the Laplace-Beltrami on surfaces. In the context of finite elements discretization for non-parametrized surfaces this method was introduced by Dziuk^34^. This finishes the construction of the variational structure behind the energy-dissipation equality $\dot{ {\mathcal E} }=-\,{\mathscr{D}}$ that holds by construction.

When one derives the partial differential equation that formally corresponds to (9), then one usually introduces an additional pressure $p:{\rm{\Omega }}\to {\mathbb{R}}$ variable that acts as a Lagrange multiplier to enforce the incompressibility condition. The resulting model for the flow of highly viscous Newtonian fluids is the coupled Stokes system

11 $$\begin{array}{rcl}-\,\nabla \cdot {{\boldsymbol{\tau }}}_{i}+\nabla {p}_{i} & = & {\bf{0}},\\ \nabla \cdot {{\bf{u}}}_{i} & = & \mathrm{0,}\end{array}$$

with shear stress ${{\boldsymbol{\tau }}}_{i}={\mu }_{i}(\nabla {{\bf{u}}}_{i}+\nabla {{\bf{u}}}_{i}^{T})$ as introduced in (6) and solved in Ω_*i*_ for $i\in \{{\rm{s}},\ell \}$ in the substrate and the liquid. The equations in the two regions are coupled by interface/boundary conditions: no-slip **u**_s_ = 0 at *z* = 0, continuity ${{\bf{u}}}_{\ell }={{\bf{u}}}_{{\rm{s}}}$ on ${{\rm{\Gamma }}}_{s,\ell }$, tangential and normal stress conditions due to surface tension at the free surfaces ${{\rm{\Gamma }}}_{s},{{\rm{\Gamma }}}_{\ell }$, and corresponding jump conditions on the interface ${{\rm{\Gamma }}}_{s,\ell }$ generated by (10). This implies a condition for the pressure-jump at the interface ${{\rm{\Gamma }}}_{{\rm{s}},\ell }$. At the contact line the Young force **f**_cl_ = 0 imposes further conditions on the triple junction using the Neumann triangle. When the velocity field is computed by solving (11), the domain Ω(*t*) is evolved according to the velocity field **u**, so that in particular the velocity **u**_Γ_ of points on the free boundary satisfy the kinematic condition

12 $$({\bf{u}}-{{\bf{u}}}_{{\rm{\Gamma }}})\cdot {\boldsymbol{\nu }}=\mathrm{0,}$$

on the free interfaces and surfaces Γ_*i*_. The fact that the domain shape Ω(*t*) is part of the unknowns makes the problem a free boundary problem. In the following we understand the evolution of the domain shapes parametrized using non-negative functions ${h}_{s}(t,x,y),{h}_{\ell }(t,x,y)$ as introduced in (3) and (4) and shown in Fig. 3. For simplicitly the dependence of ${h}_{{\rm{s}}},{h}_{\ell }$ on *y* will be dropped, since the solutions are assumed translation invariant in *y*-direction due to the particular experimental setup.

The system of Eq. (11) is now non-dimensionalized using

13 $$[x]=[z]=H,\,[t]=H{\mu }_{\ell }/{\gamma }_{{\rm{s}}},$$

and we replace the dimensional parameters by ${\tilde{\gamma }}_{{\rm{s}}}=1$, ${\tilde{\gamma }}_{\ell }={\gamma }_{\ell }/{\gamma }_{{\rm{s}}}$, ${\tilde{\gamma }}_{{\rm{s}},\ell }={\gamma }_{{\rm{s}},\ell }/{\gamma }_{{\rm{s}}}$, and $\tilde{S}=S/{\gamma }_{{\rm{s}}}$. Consequently, all experimental and numerical lengths are normalized to the initial film height $H={\bar{h}}_{\ell }$. However, when stating experimental times we rather use the scaling $t/{\bar{h}}_{\ell }$ since the multiplication with ${\bar{h}}_{\ell }$ gives a direct conversion to the physical time and the factor ${\mu }_{\ell }/{\gamma }_{{\rm{s}}}$ in (13) is constant in all the experiments. Following the standard thin-film approximation we assume that the interfaces are shallow $|{\partial }_{x}{h}_{{\rm{s}}}|\ll 1$, $|{\partial }_{x}{h}_{\ell }|\ll 1$. Then, a formal asymptotic calculation shows that *h*_s_, ${h}_{\ell }$ are solutions of a system of degenerate parabolic equations defined separately on the wetted region $\omega (t)=\{x\in {\mathbb{R}}:{h}_{\ell }(t,x) > 0\}\subset {\mathbb{R}}$ and its complement. For *x* ∈ *ω*(*t*) we have

14 $$\begin{array}{rcl}{\partial }_{t}{h}_{{\rm{s}}} & = & {\partial }_{x}({M}_{11}{\partial }_{x}{\pi }_{1}+{M}_{12}{\partial }_{x}{\pi }_{2}),\\ {\partial }_{t}{h}_{\ell } & = & {\partial }_{x}({M}_{21}{\partial }_{x}{\pi }_{1}+{M}_{22}{\partial }_{x}{\pi }_{2}),\end{array}$$

with mobility matrix ${M}_{11}={h}_{{\rm{s}}}^{3}/(3\mu )$, ${M}_{12}={M}_{21}={h}_{{\rm{s}}}^{2}{h}_{\ell }\mathrm{/(2}\mu )$, ${M}_{22}={h}_{\ell }^{3}\mathrm{/3}+{h}_{{\rm{s}}}{h}_{\ell }^{2}/\mu$, viscosity ratio $\mu ={\mu }_{{\rm{s}}}/{\mu }_{\ell }$, pressures *π*_1_ = *δE*/*δh*_s_ and ${\pi }_{2}=\delta E/\delta {h}_{\ell }$ using thin-film energy *E* obtained by expanding ${\mathcal E}$ for shallow slopes

15 $$E({h}_{\ell },{h}_{{\rm{s}}})={\int }_{\omega }\,{\tilde{\gamma }}_{\ell }(1+\tfrac{1}{2}|\nabla ({h}_{{\rm{s}}}+{h}_{\ell }{)|}^{2})+{\tilde{\gamma }}_{{\rm{s}},\ell }(1+\tfrac{1}{2}|\nabla {h}_{{\rm{s}}}{|}^{2})\,{\rm{d}}x+{\int }_{{\mathbb{R}}\backslash \omega }\,{\tilde{\gamma }}_{{\rm{s}}}(1+\tfrac{1}{2}|\nabla {h}_{{\rm{s}}}{|}^{2})\,{\rm{d}}x,$$

which explicitly gives ${\pi }_{1}=-\,({\tilde{\gamma }}_{{\rm{s}},\ell }+{\tilde{\gamma }}_{\ell }){\partial }_{xx}{h}_{{\rm{s}}}-{\tilde{\gamma }}_{\ell }{\partial }_{xx}{h}_{\ell }$, ${\pi }_{2}=-\,{\tilde{\gamma }}_{\ell }{\partial }_{xx}{h}_{{\rm{s}}}-{\tilde{\gamma }}_{\ell }{\partial }_{xx}{h}_{\ell }$. On the complement ${\mathbb{R}}\backslash \omega$ only *h*_s_ is unknown and solves the standard thin-film equation

16 $${\partial }_{t}{h}_{{\rm{s}}}={\partial }_{x}(m\,{\partial }_{x}{\pi }_{1}),$$

with degenerate mobility $m({h}_{{\rm{s}}})={h}_{{\rm{s}}}^{3}\mathrm{/(3}\mu )$ and pressure *π*_1_ = *δE*/*δh*_*s*_ = −∂_*xx*_*h*_s_. Additional boundary conditions and a kinematic conditions need to be imposed at the contact line *x*_c_ = ∂*ω*. We have ${h}_{\ell }(t,{x}_{{\rm{c}}}(t))\equiv 0$ and *h*_s_(*t*, *x*) continuous at *x*_c_, which implies for the time-derivatives the essential interface conditions

$$\begin{array}{rcl}{\dot{h}}_{\ell }+{\dot{x}}_{{\rm{c}}}\,{\partial }_{x}{h}_{\ell } & = & 0,\\ \mathop{\mathrm{lim}}\limits_{x\nearrow {x}_{{\rm{c}}}}\,{\dot{h}}_{{\rm{s}}}+{\dot{x}}_{{\rm{c}}}\,{\partial }_{x}{h}_{{\rm{s}}} & = & \mathop{\mathrm{lim}}\limits_{x\searrow {x}_{{\rm{c}}}}\,{\dot{h}}_{{\rm{s}}}+{\dot{x}}_{{\rm{c}}}\,{\partial }_{x}{h}_{{\rm{s}}},\end{array}$$

so that ${\dot{h}}_{{\rm{s}}}$, ∂_*x*_*h*_s_ are discontinuous at *x*_c_. Furthermore we require continuity of *π*_1_ and impose conservation of mass by natural interface conditions for the fluxes in (14) at *x*_c_. Contact angles at *x*_c_ appear as a natural conditions, enforced by boundary terms in the variation of the energy (15) using Reynolds transport theorem. Note that once the solution is known, we can recover the horizontal component of the flow field **u**_*i*_ = (*u*_*i*_, *w*_*i*_) in the thin-film approximation as

17 $$\begin{array}{rcl}{u}_{{\rm{s}}}(t,x,z) & = & \frac{{\partial }_{x}{\pi }_{1}(t,x)}{2\mu }{z}^{2}+{c}_{{\rm{s}}\mathrm{,1}}(t,x)\,z+{c}_{{\rm{s}}\mathrm{,2}}(t,x),\\ {u}_{\ell }(t,x,z) & = & \frac{{\partial }_{x}{\pi }_{2}(t,x)}{2}{z}^{2}+{c}_{\ell \mathrm{,1}}(t,x)\,z+{c}_{\ell \mathrm{,2}}(t,x),\end{array}$$

for *x* ∈ *ω*. The functions ${c}_{{\rm{s}}\mathrm{,1}},{c}_{{\rm{s}}\mathrm{,2}},{c}_{\ell \mathrm{,1}},{c}_{\ell \mathrm{,2}}$ depend on (*t*, *x*) and are determined using the boundary conditions *u*_s_ = 0 at *z* = 0, ${u}_{{\rm{s}}}-{u}_{\ell }={\partial }_{z}({u}_{{\rm{s}}}-\mu {u}_{\ell })=0$ at *z* = *h*_s_, ${\partial }_{z}{u}_{\ell }=0$ at $z={h}_{{\rm{s}}}+{h}_{\ell }$ as before. The flow field in the complement is determined analogously. The formal derivation of this model was performed by Kriegsmann and Miksis^20^. In order to be able to predict interface shapes for this model, we developed a novel finite-element based numerical scheme^35^, which uses advanced energetic arguments to discretize the contact line motion with the natural and essential interface conditions at *x*_c_ mentioned above.

## Discussion of Shapes and Rates

**Figure 4:**
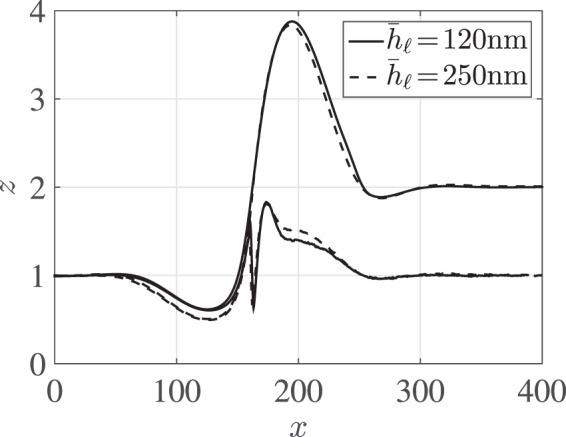
Experiments with film thickness ratio ${\bar{h}}_{\ell }:{\bar{h}}_{{\rm{s}}}\approx \mathrm{1:1}$ and different absolute film thicknesses when the rim height is about ${\rm{\max }}\,{h}_{\ell }\approx 3{\bar{h}}_{\ell }$. Dashed lines: ${\bar{h}}_{\ell }=\mathrm{(248}\pm \mathrm{2)}\,{\rm{nm}}:{\bar{h}}_{{\rm{s}}}=\mathrm{(256}\pm \mathrm{2)}\,{\rm{nm}}$, solid lines: ${\bar{h}}_{\ell }=\mathrm{(117}\pm \mathrm{3)}\,{\rm{nm}}:{\bar{h}}_{{\rm{s}}}=\mathrm{(122}\pm \mathrm{2)}\,{\rm{nm}}$.

**Figure 5:**
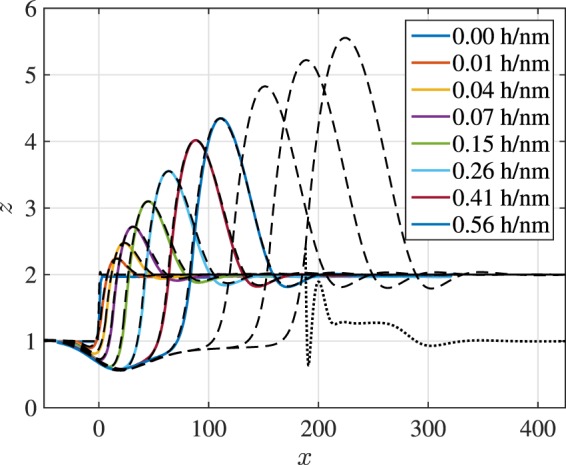
Experiments (full lines) and theory (dashed lines) for film thickness ratio ${\bar{h}}_{\ell }:{\bar{h}}_{{\rm{s}}}\approx \mathrm{1:1}$ and film thicknesses ${\bar{h}}_{\ell }=256\,{\rm{nm}}$ at different times using a series of *in*-*situ* scans of upper PS-air and PMMA-air interface. The three additional theoretical profiles are at *t* = 0.84, 1.13, 1.41 h/nm, where the dotted line shows the PS-PMMA of the latest profile.

**Figure 6:**
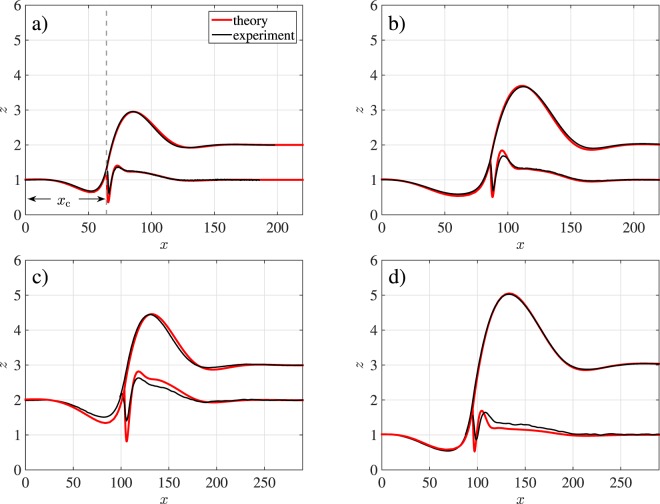
Overlap of experimental and theoretical interfaces for different thickness ratios ${\bar{h}}_{\ell }:{\bar{h}}_{{\rm{s}}}$ and dewetting times *t* at *T* = 140 °C. Rim profiles are chosen at times *t* where in (**a**,**c**,**d**) ${\rm{\max }}\,{h}_{\ell }\approx 2\,{\bar{h}}_{\ell }$ and in (**b**) ${\rm{\max }}\,{h}_{\ell }\approx 3\,{\bar{h}}_{\ell }$. (**a**,**b**) ${\bar{h}}_{\ell }:{\bar{h}}_{{\rm{s}}}\approx \mathrm{1:1}=\mathrm{(248}\pm \mathrm{2)}\,{\rm{nm}}\mathrm{:(256}\pm \mathrm{2)}\,{\rm{nm}}$ at times (**a**) *t* = 0.13 h/nm and (**b**) *t* = 0.42 h/nm, (**c**) ${\bar{h}}_{\ell }:{\bar{h}}_{{\rm{s}}}\approx \mathrm{1:2}=\mathrm{(47}\pm \mathrm{1)}\,{\rm{nm}}:\,\mathrm{(90}\pm \mathrm{2)}\,{\rm{nm}}$ at *t* = 0.09 h/nm, (**d**) ${\bar{h}}_{\ell }:{\bar{h}}_{{\rm{s}}}\approx \mathrm{2:1}=\mathrm{(89}\pm \mathrm{2)}\,{\rm{nm}}:\,\mathrm{(44}\pm \mathrm{2)}\,{\rm{nm}}$ at *t* = 0.18 h/nm. Experimental profiles are averaged over 30 scan lines of a straight front.

For a fixed thickness ratio (13) shows that, due to the absence of other intrinsic time and length scales for Newtonian liquids, the influence of the absolute height is to scale time proportionally without changing the rescaled rim profiles. To check this prediction two experimental liquid-liquid systems with thickness ratio ${\bar{h}}_{\ell }:{\bar{h}}_{{\rm{s}}}=\mathrm{1:1}$ but absolute film thicknesses ${\bar{h}}_{\ell }\approx 100\,{\rm{nm}}$ and ${\bar{h}}_{\ell }\approx 250\,{\rm{nm}}$ were observed. An overlap of the emerged rim profiles is shown in Fig. 4 for corresponding dewetting distances. The good reproducibility of the characteristic rim profiles within experimental errors confirms the previously made assumption that the fluids can be considered Newtonian and allows us to focus our study on different aspect ratios. Comparing experimentally measured and theoretically computed interface profiles, we find an excellent agreement of both the characteristic shapes of the liquid-air/substrate-air interfaces measured by *in*-*situ* AFM in Fig. 5 and the full profile when combining the upper interfaces with the AFM measurement of the burried liquid-liquid interface obtained for selected experiments after removing the PS layer in Fig. 6(a–d). The material of the dewetting liquid (PS) accumulates in a rim which, by conservation of mass, grows in time when the liquid retracts from the substrate (PMMA). Away from the rim the interfaces decay in an oscillatory fashion into their prepared constant states ${h}_{{\rm{s}}}(t,x),{h}_{\ell }(t,x)\to {\bar{h}}_{{\rm{s}}},{\bar{h}}_{\ell }$. Also some material of the liquid substrate is dragged along generating a depletion near *x* < *x*_c_ and an accumulation of substrate material near *x* > *x*_c_. The contact line itself is elevated by the flow, a dynamic feature quite common for soft substrates^36^ but not observed in stationary droplets for sufficiently thick substrates^17^. Right next to the contact line, the liquid-liquid interface extends deeply into the substrate and generates a trench which generates additional resistance against the dewetting motion. The size of this trench depends only weakly on the size of the dewetting rim, *i*.*e*., the dewetting distance.

Compared to the ratio ${\bar{h}}_{\ell }:{\bar{h}}_{{\rm{s}}}=\mathrm{1:1}$ in Fig. 6(a,b), thickness ratios of 2:1 or 1:2 do not lead to qualitatively new features. For smaller aspect ratio ${h}_{\ell }:{h}_{{\rm{s}}}=\mathrm{1:2}$, cf. Fig. 6(c), the above described characteristic features of the rim profile grow and for bigger aspect ratio ${h}_{\ell }:{h}_{{\rm{s}}}=\mathrm{2:1}$, cf. Fig. 6(d), these features shrink in size slightly. For ${\bar{h}}_{{\rm{s}}}\to 0$ we expect to observe shapes similar to that of a film dewetting from a solid substrate. The match of experiment and simulation is in all cases almost perfect, within the limits of reproducibility that can be estimated from Fig. 4.

**Figure 7:**
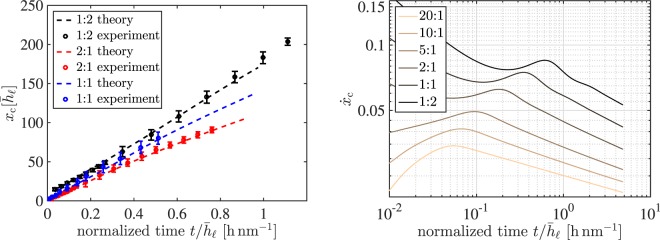
(left) Non-dimensional dewetted distance *x*_c_ for thickness ratios ${\bar{h}}_{\ell }:{\bar{h}}_{{\rm{s}}}\approx \mathrm{1:1}=\mathrm{(248}\pm \mathrm{2)}$ ${\rm{nm}}\mathrm{:(256}\pm \mathrm{2)}\,{\rm{nm}}$ and ${\bar{h}}_{\ell }:{\bar{h}}_{{\rm{s}}}\approx \mathrm{2:1}=\mathrm{(89}\pm \mathrm{2)}\,{\rm{nm}}\mathrm{:(44}\pm \mathrm{2)}\,{\rm{nm}}$ and ${\bar{h}}_{\ell }:{\bar{h}}_{{\rm{s}}}\approx \mathrm{1:2}=\mathrm{(47}\pm \mathrm{1)}\,{\rm{nm}}\mathrm{:(90}\pm \mathrm{2)}\,{\rm{nm}}$ from experiment and numerical simulation suggesting constant dewetting rates ${\dot{x}}_{{\rm{c}},\mathrm{1:1}}\approx 4.4\cdot {10}^{-2}\,{\rm{nm}}/{\rm{s}}$ and ${\dot{x}}_{{\rm{c}},\mathrm{2:1}}\approx 3.0\cdot {10}^{-2}\,{\rm{nm}}/{\rm{s}}$ and ${\dot{x}}_{{\rm{c}}\mathrm{,1:2}}\approx 5.2\cdot {10}^{-2}\,{\rm{nm}}/{\rm{s}}$, where longer simulation times (right) reveal that rates ${\dot{x}}_{{\rm{c}}}$ decrease depending on details such as the aspect ratio.

For small dewetting distances, the dewetting rates in Fig. 7 suggest a linear behavior *x*_c_ ~ *t* for all thickness ratios in agreement with the results by Lambooy and coworkers^24^. For fixed substrate film thickness ${\bar{h}}_{{\rm{s}}}$, the dewetting rates are larger for liquid layers thinner than the substrate, ${\bar{h}}_{\ell } < {\bar{h}}_{{\rm{s}}}$, and smaller for thicker liquid layers, ${\bar{h}}_{\ell } > {\bar{h}}_{{\rm{s}}}$. But, a close inspection of the seemingly constant dewetting rates in Fig. 7 (left) indicates that the dewetting velocity slowly decreases over time. This fact is most apparent for aspect ratio 2:1, while for aspect ratio 1:2 the velocity even appears to increase. However, the experimental accuracy is not sufficient to fully clarify this claim.

To clarify the dependence of the dewetting rates, results from simulation are plotted in Fig. 7 (right) for physical dewetting times of several month, which are not accessible experimentally together with further results for other film thickness ratios. Note the small variation in the velocities during dewetting, which explains why dewetting rates appear almost constant. However, the intricate transient behavior of the velocity ${\dot{x}}_{{\rm{c}}}$ featuring inflection points in the simulations coincides with the before mentioned experimental observation. For instance, for an aspect ratio of 2:1 and the experimentally accessible (normalized) times *t* = 10^−1^ … 10^0^ h nm^−1^, cf. Fig. 7 (left), the dewetting rate decreases, while for an aspect ratio of 1:2 the rate slightly increases within the observed dewetting interval. Note the striking agreement of all experimental and theoretical rescaled rates *x*_c_(*t*) also suggests the validity of the introduced parameters, *i*.*e*., viscosities and surface tensions. Furthermore, for all simulated parameters we find that for large times the velocity slowly decays to zero. Next, we are going to discuss the physical dissipation mechanism behind the observed transient dewetting rates.

## Discussing the Role of Dissipation

**Figure 8:**
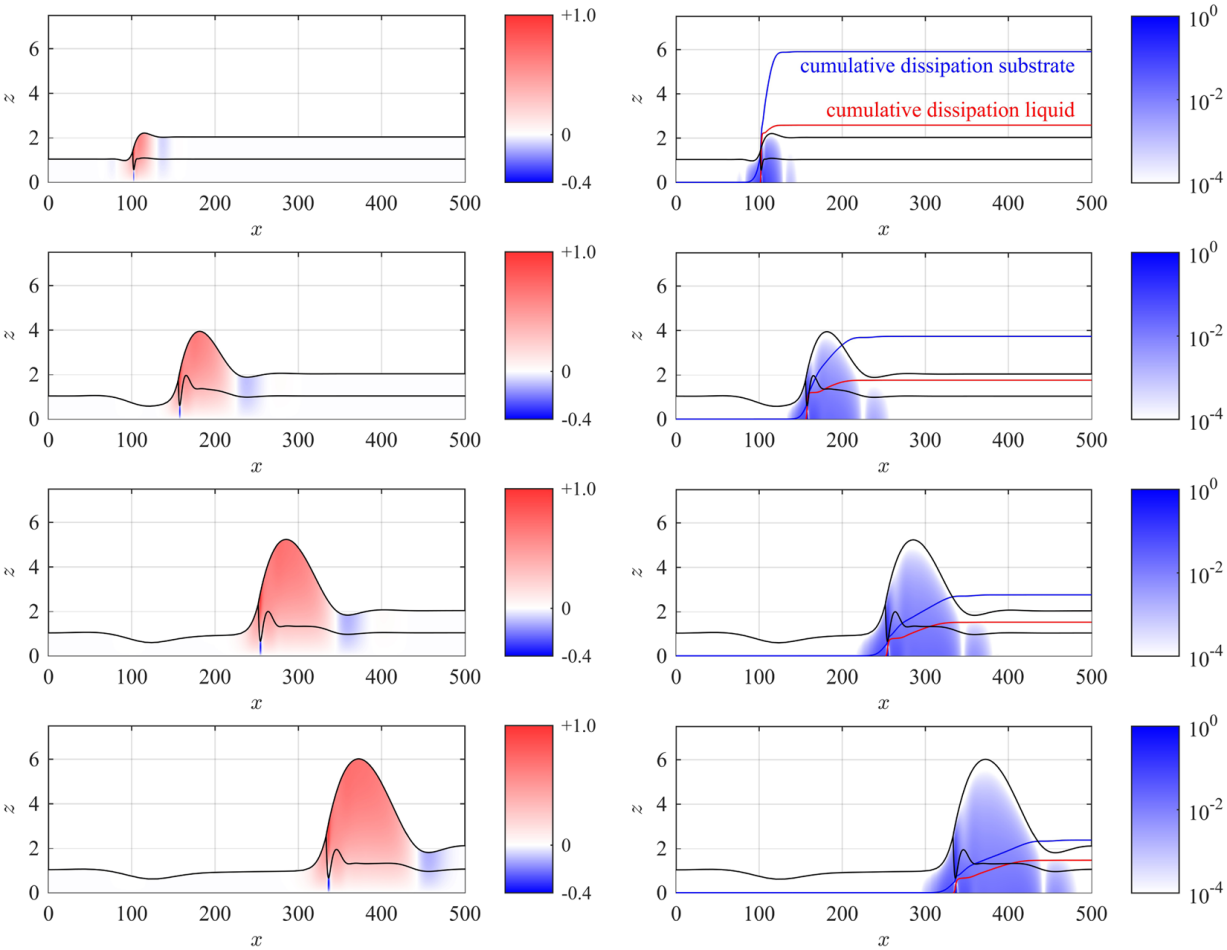
(left) Flow fields in the liquid substrate *u*_s_(*t*, *x*, *z*) and in the dewetting liquid ${u}_{\ell }(t,x,z)$ and (right) the corresponding energy dissipation *D*_s_(*t*, *x*, *z*) = (∂_*z*_*u*_s_(*t*, *x*, *z*))^2^ and ${D}_{\ell }(t,x,z)=\mu {({\partial }_{z}{u}_{\ell }(t,x,z))}^{2}$ on a logarithmic scale normalized to their respective maximal values computed numerically from the thin-film model during dewetting of liquid-liquid system with aspect ratio 1:1 at times *t* = 0.005, 0.36, 1.07, 1.77 h/nm increasing from top to bottom. The addidional curves in the right panel show the cumulative dissipation in the substrate ${\int }_{-\infty }^{x}\,{\int }_{0}^{{h}_{{\rm{s}}}}\,{D}_{{\rm{s}}}{\rm{d}}z\,{\rm{d}}x$ (blue line) and in the liquid ${\int }_{{x}_{{\rm{c}}}}^{x}\,{\int }_{{h}_{{\rm{s}}}}^{{h}_{{\rm{s}}}+h}\,{D}_{\ell }{\rm{d}}z\,{\rm{d}}x$ (red line) normalized with an arbitrary but time-independent constant.

Since the thin-film model accurately predicts shapes and speeds of the liquid-liquid dewetting, we extend our approach and discuss local flow features that are experimentally inaccessible in order to explain the observed dewetting dynamics. For instance, the rescaled dissipation balances with the driving surface tension in a 2-*D* cross section according to

18 $$\tilde{{\mathscr{D}}}={\int }_{{{\rm{\Omega }}}_{{\rm{s}}}}\,\tfrac{1}{2}{({\partial }_{z}{u}_{{\rm{s}}})}^{2}\,{\rm{d}}x\,{\rm{d}}z+{\int }_{{{\rm{\Omega }}}_{\ell }}\,\tfrac{\mu }{2}{({\partial }_{z}{u}_{\ell })}^{2}\,{\rm{d}}x\,{\rm{d}}z=-\,\tilde{S}\times {\dot{x}}_{{\rm{c}}},$$

with rescaled spreading coefficient $\tilde{S}={\tilde{\gamma }}_{{\rm{s}}}-({\tilde{\gamma }}_{{\rm{s}},\ell }+{\tilde{\gamma }}_{\ell })$. While the driving force is straightforward to understand, the dissipation depends on local details of the flow field. As a representative example to discuss the qualitative behavior we use numerical solutions with thickness ratio 1:1 and show rim profiles at different times overlapped with the dominant horizontal component of the velocity reconstructed using (17) in the left panel of Fig. 8 and the corresponding dissipation ${({\partial }_{z}{u}_{\ell })}^{2}$ and *μ*(∂_*z*_*u*_s_)^2^ in the right panel. This gives an unprecedented insight to where the flow is resisting to the driving force.

The flow fields in the left panels of Fig. 8 point mainly in the positive *x*-direction with its maximum at the contact line. Away from the rim $|x-{x}_{{\rm{c}}}|\gg 0$ the flow field vanishes. Below the depression of the liquid-substrate interface there is a rather strong and localized backflow in the liquid substrate. This backflow is created due to conservation of mass, which forces the flow in the substrate to balance the forward transport of the depression. Due to the boundary conditions ${\partial }_{z}{u}_{{\rm{s}},\ell }=0$ the dissipation vanishes at the liquid/air and substrate/air interfaces, whereas the flow field is zero at the solid/substrate interface *z* = 0. The latter results in a large shear rate and a large energy dissipation at the solid interface, cf. right panels of Fig. 8. Close to the backflow and close to the contact line the maximal dissipation density is reached. However, due to the small size of these regions the integrated dissipation near the contact line and in the remaining rim are of the same order, at least for the transient times and moderately large rims considered here. To visualize this fact, we additionally show the cumulative dissipation inside the (liquid) substrate and inside the (dewetting) liquid in Fig. 8 for different times. Since the shear rate is large at the solid interface where *z* = 0, clearly the dissipation for an aspect ratio 1:1 is large in the substrate for the short and intermediate times considered experimentally. Nevertheless, with the volume of the liquid rim increasing in time, ultimately the dissipation in the liquid layer will dominate for large times or for higher aspect ratios. A slightly more detailed visual description of this dynamics and the corresponding experiments is provided in the attached supplemental video.

Accordingly, one can identify two different zones where the energy is dissipated in the liquid and the substrate. A significant amount of the dissipation is produced in a small region near the contact line. This can be seen in the steep increase of the cumulative dissipation in the right panels of Fig. 8. The remaining contribution to the dissipation is more or less evenly distributed over the rim width resulting in a moderate and constant increase of the cumulative dissipation over the width of the rim. For large times this bulk contribution will dominate the dissipation and forces the velocity to decay to zero. This can also be seen in the temporal evolution of the dissipation profiles, which is decreasing due to the quadratic dependence on the velocity scale in (18). This variable contribution to the energy dissipation $\tilde{{\mathscr{D}}}$ directly impacts the observed dewetting rates. This can be best explained on the basis of the known dewetting behavior on solid substrates, which qualitatively also applies for dewetting from liquid substrates. When the contact line position is *x*_c_(*t*) and starts at *x*_c_(*t* = 0) = 0, then asymptotically the rim cross-sectional area grows proportionally to ${x}_{{\rm{c}}}\times {h}_{\ell }$ by volume uptake from the unperturbed liquid layer. Consequently, assuming self-similar growth, typical rim geometry such as rim width or like rim height grow proportionally to $\sqrt{{x}_{{\rm{c}}}}$. Additionally, we assume self-similar growth of the dissipation according to

19 $$\tilde{{\mathscr{D}}}({\bf{u}})\sim {({x}_{{\rm{c}}})}^{\alpha }\,{\dot{x}}_{{\rm{c}}}^{2},$$

where it remains to specify *α* using the dominating dissipation mechanism and its dependence on rim geometry. In the intermediate slip model^11^, the dominant contribution comes from a substrate dissipation, so that the total dissipation is proportional to the rim width and thereby *α* = 1/2 in (19). Then, the energy-dissipation balance produces ${({x}_{{\rm{c}}})}^{\alpha }\,{\dot{x}}_{{\rm{c}}}^{2}\sim -\,S\times {\dot{x}}_{{\rm{c}}}$ leading to an asymptotic *x*_c_(*t*) ~ *t*^*β*^ dewetting law with *β* = 2/3. Another example^11^ is the no-slip model, where the dissipation is predominantly localized near the contact line and in the bulk domain. The contact line area does not scale with the volume, whereas the gradients in the dissipation in (18) cancel the growth of the cross-sectional area, thereby both leading to *α* = 0 in (19). This produces a linear dewetting law *β* = 1, however with logarithmic corrections^37^. Similarly, the power-law dewetting rates on liquid substrates predicted by Brochard *et al*.^14^ rely on the assumptions that the dissipation is generated in only one such localized zone together with a nearly self-similar growth of rim shapes. However, these assumptions fail in the considered situation of liquid-liquid dewetting since dissipation is clearly not generated in one single zone but accumulates in the substrate, in the liquid and near contact lines on a similar order of magnitude. This explains why in our setting the liquid-liquid dewetting process is not in a regime dominated by a specific dissipation mechanism that would admit a simplification to a power-law rate, and thereby challenges the applicability of the theoretical results by Joanny^13^ and Bochard-Wyart *et al*.^14^ to experimental systems considered in this paper in early stages. However, the weak scaling of the dissipation with increasing *x*_c_ theoretically explains the nearly linear dewetting rate, that was observed experimentally by Krausch^24^ and in the present work.

The consideration of the liquid-liquid dewetting using thin film models with explicit contact line dynamics, conducted here, allows to describe the variable energy dissipation in a liquid-liquid system and to quantitatively derive rim shapes and dewetting rates. Nevertheless, the predicted slowdown of the dewetting velocity is expected when the dissipated energy is not soley confined to the contact line. The exact slopes in the log-log plot of Fig. 7 (right) depend on details such as thickness ratio and viscosities, and thereby do not support a universal power-law behavior. This observation confirms previous speculations by Krausch *et al*.^24^ that were based on experimental findings about the transient nature of the experimentally measured dewetting dynamics.

## Conclusion

Motivated by the long-standing puzzle between theoretically predicted and experimentally observed rates for liquid-liquid dewetting, we performed a combined theoretical and experimental investigation of the transient interface shapes and dewetting rates. Conducting a full simulation of the sharp interface thin-film model for Newtonian liquids without any a priori assumptions on rim shape development or energy dissipation we obtained a full agreement with experimentally determined interface shapes and dewetting dynamics using the relevant experimental parameters like viscosities, aspect ratios, and surface energies. As the main tool to assess the transient nature of the flow, we reconstructed local flow and dissipation fields. Such a detailed analysis of a local energy balance provides deep insights into underlying mechanisms driving such a process.

By analyzing the local energy dissipation, we have found that the liquid-liquid dewetting system is in a transient state with no self-similar behavior and the dissipation is not distributed exclusively at the contact line, in the substrate, or in the bulk. While the dewetting rate in the observed experimental regime is almost constant and thereby of powerlaw type in the strict sense, the absence of self-similarity and localization of dissipation underlines the absence of a dominant mechanism behind this rate. A similar energetic argument provided the explanation why, for very large times beyond experimental reach, the dewetting velocity slowly decreases to zero. Such predictions would be impossible using heuristic approaches, since the transient internal flow is rather complex and results from a complex interaction of substrate and liquid. Without such a theoretical toolbox, the observed dewetting rates might otherwise be misinterpreted as a regime with potential dominant physical effects. The demonstrated ability to use energetic arguments to quantitatively describe liquid-liquid systems thus set grounds for a similarly complete understanding as already obtained for liquid-solid dewetting systems. In particular, the analysis of the local dissipation distribution provides a powerful tool to identify dominant physical regimes or their absence. It might be in particular possible to extend this approach also to fluids with complex rheological behavior.

## Electronic supplementary material

Supplemental movie

## References

[1] Grmela M, Öttinger H (1997). Dynamics and thermodynamics of complex fluids. I. Development of a general formalism. Physical Review E.

[2] Morrison P (1998). Hamiltonian description of the ideal fluid. Reviews of Modern Physics.

[3] de Groot, S. & Mazur, P. *Non-equilibrium thermodynamics* (North-Holland, 1962).

[4] Otto F (2001). The geometry of dissipative evolution equations: the porous medium equation. Communications in Partial Differential Equations.

[5] Peletier, M. Variational modelling: Energies, gradient flows, and large deviations. *arXiv preprint arXiv:1402*.*1990* (2014).

[6] Helmholtz HV (1868). Zur Theorie der stationären Ströme in reibenden Flüssigkeiten. Wiss. Abh..

[7] Strutt J (1871). Some general theorems relating to vibrations. Proceedings of the London Mathematical Society.

[8] Korteweg D (1883). XVII. On a general theorem of the stability of the motion of a viscous fluid. Philosophical Magazine.

[9] Gentili D, Foschi G, Valle F, Cavallini M, Biscarini F (2012). Applications of dewetting in micro and nanotechnology. Chemical Society Reviews.

[10] Oron A, Davis S, Bankoff S (1997). Long-scale evolution of thin liquid films. Reviews of Modern Physics.

[11] Fetzer R, Jacobs K, Münch A, Wagner B, Witelski T (2005). New slip regimes and the shape of dewetting thin liquid films. Physical Review Letters.

[12] Craster R, Matar O (2009). Dynamics and stability of thin liquid films. Reviews of Modern Physics.

[13] Joanny J (1987). Wetting of a liquid substrate. Physicochemical Hydrodynamics.

[14] Brochard-Wyart F, Martin P, Redon C (1993). Liquid/liquid dewetting. Langmuir.

[15] Bonn D, Eggers J, Indekeu J, Meunier J, Rolley E (2009). Wetting and spreading. Reviews of Modern Physics.

[16] Craster R, Matar O (2006). On the dynamics of liquid lenses. Journal of Colloid and Interface Science.

[17] Bommer S (2013). Droplets on liquids and their journey into equilibrium. The European Physical Journal E.

[18] Pototsky A, Bestehorn M, Merkt D, Thiele U (2004). Alternative pathways of dewetting for a thin liquid two-layer film. Physical Review E.

[19] Fisher L, Golovin A (2005). Nonlinear stability analysis of a two-layer thin liquid film: Dewetting and autophobic behavior. Journal of Colloid and Interface Science.

[20] Kriegsmann J, Miksis M (2003). Steady motion of a drop along a liquid interface. SIAM Journal on Applied Mathematics.

[21] Jachalski S, Peschka D, Münch A, Wagner B (2014). Impact of interfacial slip on the stability of liquid two-layer polymer films. Journal of Engineering Mathematics.

[22] Pototsky A, Bestehorn M (2016). Faraday instability of a two-layer liquid film with a free upper surface. Physical Review Fluids.

[23] Karapetsas G, Craster R, Matar O (2011). Surfactant-driven dynamics of liquid lenses. Physics of Fluids.

[24] Lambooy P, Phelan K, Haugg O, Krausch G (1996). Dewetting at the liquid-liquid interface. Physical Review Letters.

[25] Wang C, Krausch G, Geoghegan M (2001). Dewetting at a polymer-polymer interface: film thickness dependence. Langmuir.

[26] Pan Q, Winey K, Hu H, Composto R (1997). Unstable polymer bilayers. 2. The effect of film thickness. Langmuir.

[27] McGraw J, Salez T, Bäumchen O, Raphaël E, Dalnoki-Veress K (2012). Self-similarity and energy dissipation in stepped polymer films. Physical Review Letters.

[28] Salez T (2012). Numerical solutions of thin-film equations for polymer flows. The European Physical Journal E.

[29] Wu S (1970). Surface and interfacial tensions of polymer melts. II. Poly(methyl methacrylate), poly(*n*-butyl methacrylate), and polystyrene. The Journal of Physical Chemistry.

[30] Hirai Y, Yoshikawa T, Takagi N, Yoshida S, Yamamoto K (2003). Mechanical properties of poly-methyl methacrylate (PMMA) for nano imprint lithography. Journal of Photopolymer Science and Technology.

[31] Lomellini P (1992). Effect of chain length on the network modulus and entanglement. Polymer.

[32] Morozov A, van Saarloos W (2007). An introductory essay on subcritical instabilities and the transition to turbulence in visco-elastic parallel shear flows. Physics Reports.

[33] Ambrosio, L., Fusco, N. & Pallara, D. *Functions of bounded variation and free discontinuity problems*, vol. 254 (Clarendon Press Oxford, 2000).

[34] Dziuk G (1990). An algorithm for evolutionary surfaces. Numerische Mathematik.

[35] Huth R, Jachalski S, Kitavtsev G, Peschka D (2015). Gradient flow perspective on thin-film bilayer flows. Journal of Engineering Mathematics.

[36] Style R (2013). Universal deformation of soft substrates near a contact line and the direct measurement of solid surface stresses. Physical Review Letters.

[37] Münch A, Wagner B, Witelski TP (2005). Lubrication models with small to large slip lengths. Journal of Engineering Mathematics.

